# Maternal mortality in the covid-19 pandemic: findings from a rapid systematic review

**DOI:** 10.1080/16549716.2021.1974677

**Published:** 2022-04-04

**Authors:** Clara Calvert, Jeeva John, Farirai P Nzvere, Jenny A. Cresswell, Sue Fawcus, Edward Fottrell, Lale Say, Wendy J. Graham

**Affiliations:** a Centre for Global Health, Usher Institute, University of Edinburgh, UK; b Department of Population Health, London School of Hygiene and Tropical Medicine, UK; c UNDP-UNFPA-UNICEF-WHO-World Bank Special Programme of Research, Development and Research Training in Human Reproduction (HRP), Department of Sexual and Reproductive Health and Research, World Health Organization, Geneva, Switzerland; d Department of Obstetrics and Gynaecology, University of Cape Town, Rondebosch, South Africa; e UCL Institute for Global Health, Faculty of Population Health Sciences, University College London, London, UK; f Department of Infectious Disease Epidemiology, London School of Hygiene and Tropical Medicine, UK

**Keywords:** Maternal deaths, maternal mortality ratio, rapid systematic review, Sars-Cov-2

## Abstract

**Background:**

The COVID-19 pandemic is having significant direct and associated effects on many health outcomes, including maternal mortality. As a useful marker of healthcare system functionality, trends in maternal mortality provide a lens to gauge impact and inform mitigation strategies.

**Objective:**

To report the findings of a rapid systematic review of studies on levels of maternal mortality before and during the COVID-19 pandemic.

**Methods:**

We systematically searched for studies on the 1st March 2021 in MEDLINE and Embase, with additional studies identified through MedRxiv and searches of key websites. We included studies that reported levels of mortality in pregnant and postpartum women in time-periods pre- and during the COVID-19 pandemic. The maternal mortality ratio was calculated for each study as well as the excess mortality.

**Results:**

The search yielded 3411 references, of which five studies were included in the review alongside two studies identified from grey literature searches. Five studies used data from national health information systems or death registries (Mexico, Peru, Uganda, South Africa, and Kenya), and two studies from India were record reviews from health facilities. There were increased levels of maternal mortality documented in all studies; however, there was only statistical evidence for a difference in maternal mortality in the COVID-19 era for four of these. Excess maternal mortality ranged from 8.5% in Kenya to 61.5% in Uganda.

**Conclusions:**

Measuring maternal mortality in pandemics presents many challenges, but also essential opportunities to understand and ameliorate adverse impact both for women and their newborns. Our systematic review shows a dearth of studies giving reliable information on levels of maternal mortality, and we call for increased and more systematic reporting of this largely preventable outcome. The findings help to highlight four measurement-related issues which are priorities for continuing research and development.

## Background

The COVID-19 pandemic has highlighted enormous differentials in vulnerability and fragility, at both individual and health system levels, and within and between countries[1]. These patterns have emerged from research, surveillance and evidence syntheses conducted rapidly, revealing clinical, racial and ethnic groups at increased risk of severe infection and poor outcomes [2]. Preventive and treatment strategies are being implemented, albeit with huge variations in reach and fidelity between health system settings depending on resource limitations and political expediency, as well as due to the evolving scientific evidence on the most effective solutions. Assessing the impact of COVID-19 at a population level requires not only consideration of the direct effects of COVID-19 but also the indirect – or so-called collateral or side effects – arising from disruption to routine services in terms of availability, access and quality of care [3].

One of the most prevalent health-related states facing this double jeopardy and found in every country around the world is pregnancy. With an estimated 213 million women becoming pregnant each year, resulting in approximately 140 million deliveries, the projected magnitude of impact from COVID-19, both directly through the known risks of mortality from severe disease and indirectly through disruption to health services, is enormous [4–6]. Numerous studies have reported the case-fatality for COVID-19 among pregnant and postpartum women. Allotey and colleagues estimated that 0.02% of pregnant women with confirmed COVID-19 died based on data from 59 studies [7], although there was wide variation in individual study estimates. Vargara-Merino and colleagues, for example, found figures varying between 0% and 11.1% across different systematic reviews [8]. These estimates, however, all fail to capture the indirect impacts of COVID-19 on maternal mortality. Adding into the equation the impact of this disruption on neonates, the true scale of potential damage from the pandemic becomes evident [9].

So, what is known reliably about population levels of maternal mortality since the emergence of COVID-19? Although measuring these deaths routinely is notoriously difficult, even in the presence of complete vital registration, the rapid set-up of new, and enhancement of existing, surveillance systems to track the pandemic has had the potential to help fill data gaps [10]. Have these measurement opportunities been seized wherever possible or are we seeing a repeat of the data void seen over two-decades ago with the emergence of HIV/AIDS impacting on maternal death? [11] The main aim of this paper is to report the findings of a rapid systematic review of evidence on levels of maternal mortality before and since the onset of the COVID-19 pandemic, with a particular focus on the methodological quality of these studies. The findings are used to highlight key gaps relevant to understanding the direct and associated impact of the pandemic on maternal death and service utilisation, and to improving future reporting of maternal mortality. In the Discussion, we integrate four text panels to highlight specific measurement issues emerging from the review and featuring strongly in the life-works of the late Professor Peter Byass, to whom this Journal Special Edition is dedicated.

## Methods

The systematic review was conducted in line with the recommendations of the PRISMA guidelines (Appendix S1)[12]. The study protocol was registered with PROSPERO (Record: CRD42020219889).

### Search strategy

In collaboration with an experienced librarian, we developed a search strategy combining terms for ‘maternal’ and ‘mortality’ to identify studies across two databases – MEDLINE and Embase. This was adapted from a search strategy previously developed by the World Health Organization to identify studies reporting on maternal mortality [13]. A simplified search was also conducted in MedRxiv which contains pre-peer review articles. The search terms are provided in Appendix S2. Searches were conducted on 1 March 2021, and limited to studies published from 1 January 2020. There were no language restrictions.

Reference lists of included studies were reviewed for any additional relevant articles. We additionally searched for grey literature, using the same terms as for the reference databases, on maternal mortality, on UN agency websites, such as WHO and UNFPA, as well as those of professional bodies (such as FIGO), the US Centers for Disease Control, and the John Hopkins University dedicated repository for research on COVID-19, maternal and child health (see AppendixS 2).

### Inclusion criteria

We included randomised controlled trials, repeated cross-sectional studies, cohort studies or time series studies that reported levels of mortality in pregnant and postpartum women in both the pre- and during COVID-19 pandemic time period. We included studies that captured the COVID-19 pandemic as any time period since WHO declared the outbreak a Public Health Emergency of International Concern (30 January 2020); however, we also included any studies reporting key outcomes from 1 January 2020 as covering the COVID-19 era if the majority of the study period was following 30 January 2020. The pre-pandemic time period was accepted as defined by the study authors.

Outcomes of interest were the: (1) maternal mortality ratio; (2) maternal mortality rate; (3) percentage of deaths to women of reproductive age that were maternal; (4) magnitude of association between key socio-demographic characteristics and facility type and maternal mortality and; (5) distribution of causes of maternal death. Studies were included regardless of the definition of maternal mortality used, covering direct and indirect obstetric deaths as well as pregnancy-related deaths (any death to a pregnant or postpartum women regardless of cause of death) [14], but any studies which only had provided estimates that included maternal deaths to women beyond one year postpartum were excluded.

Studies were excluded if they only included specific sub-groups of pregnant and postpartum women (e.g. only adolescents or women with a specific medical condition such as diabetes or HIV). As we were interested in the overall impact of COVID-19 on maternal mortality, we also excluded any studies which were restricted to only women with suspected and/or confirmed COVID-19.

### Study selection

References identified from each database were imported into EndNote 9 and de-duplicated. They were then reviewed by two of the study authors using Raayan (https://www.rayyan.ai/), and disagreements resolved by a third author. References which met the title/abstract criteria applied under initial screening then moved forward to full-text screening. One of the study authors applied the full-text screening eligibility criteria, with a second author applying the same criteria to a randomly-selected 50% sample of these references. Disagreements were resolved by discussion. The entire study selection process was carried out by four of the authors (CC, JJ, FN, WJG).

### Data extraction

The following data were extracted from articles included in the review by one of the study authors using Microsoft Excel: year of publication; study setting; study population; study design; observation period in pre-COVID era and in COVID-19 era; levels and causes of maternal mortality (disaggregated by time period). Where pre-COVID-19 data on maternal mortality were available for multiple different time periods pre-pandemic, for example annual estimates since 2015, we extracted estimates just from 2019. The data extraction was double checked by a second author. We contacted the study authors for additional information where needed.

### Risk of bias assessment

We undertook an assessment of risk of bias, with each study assessed as high, low or unclear risk of bias for the following domains: (1) extent to which study population is geographically representative of the entire population; (2) extent to which study populations include women that had either facility or home deliveries; (3) definition of maternal deaths; (4) definition of denominator; (5) alignment of COVID-19 time period with emergence of COVID-19 or lockdown; (6) comparability of pre- and post-COVID-19 study populations. The final domain was assessed by looking at whether there was a decrease in the number of births or pregnancies documented in the COVID-19 period compared with the pre-COVID-19 period; given the early stage of the pandemic which this review has captured, fertility rates will not yet have been impacted and therefore any increase or decrease will likely reflect either changes in recording of births or deaths, or changes in where women are delivering. If there was no change or change less than 10%, the study was considered at low risk of bias. Full details of the criteria by which studies were classified as at high risk of bias for each of the domains are provided in Supplementary Table S1 (Appendix S3).

### Synthesis of results

We calculated and summarised the maternal mortality ratio for each study as: (1) the number of maternal deaths per 100,000 live births and; (2) the number of maternal deaths per 100,000 deliveries. We then calculated the change in the maternal mortality ratio first by subtracting the maternal mortality ratio pre-COVID-19 from the maternal mortality ratio in the COVID-19 period, and expressing this as a percentage of the maternal mortality ratio in the COVID-19 period.

The maternal mortality rate was calculated as the number of maternal deaths per 100,000 women of reproductive age, and cause of death was reported as presented in the study.

Due to heterogeneity between the study populations and study methods we did not calculate any pooled measure of effect.

## Results

### Search strategy

**Figure 1.**
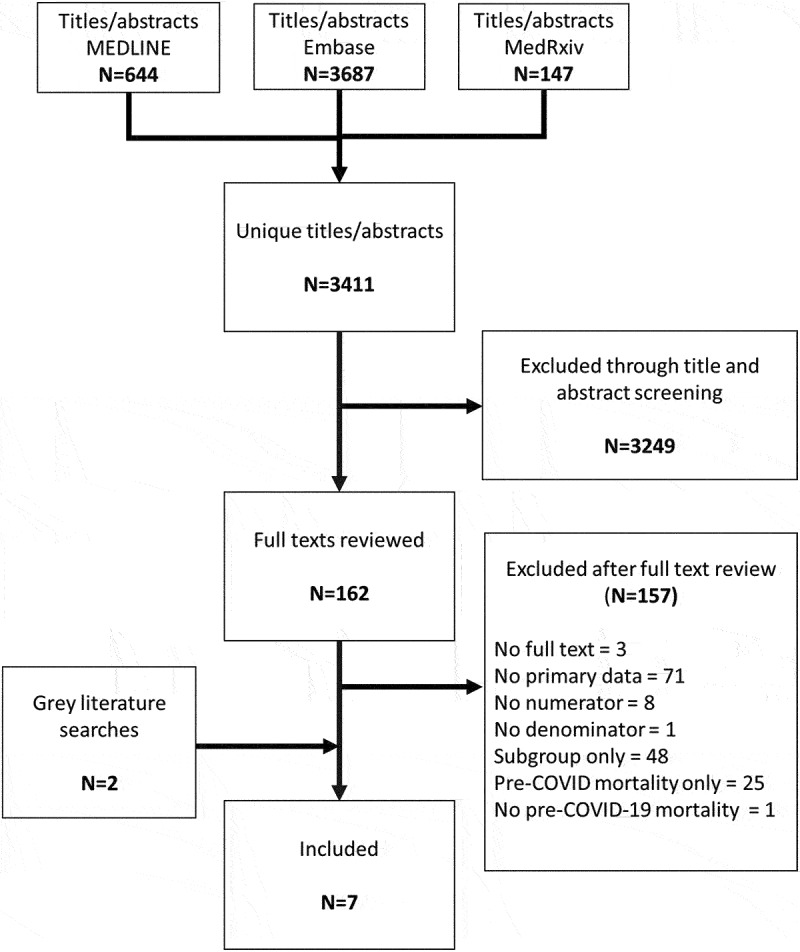
Systematic review study identification.

Figure 1 provides a detailed overview of the study identification and selection process. The literature search strategy yielded 3411 titles and abstracts, of which 162 titles and abstracts on maternal mortality were included for full-text review. Of these full texts, 157 were excluded, primarily because they did not provide primary data (N = 71) or because they only looked at maternal mortality in a subgroup of women (N = 48). Within this latter group of excluded studies, most were examining levels of maternal mortality only amongst women who had suspected or confirmed COVID-19. The grey literature searches identified a further two studies, giving a total of seven studies for inclusion. Additional data were provided by the authors of one study [15] and a report with updated results was provided by the authors of another study [16,17].

### Study description

Table 1 provides an overview of the included studies. The seven included studies come from six countries – one each from Mexico [18], Peru [19], Uganda [15], South Africa [17] (with additional reported data [16]) and Kenya [20], and two studies from India [21,22]. The source of data on maternal mortality varied between the studies, with three drawing on data from national health information systems or death registries [15,17,20], one study drawing on weekly epidemiological reports from the Ministry of Health [18] and one study using data from the national death registry information system [19]. The two remaining studies, both from India, did not explicitly state the data source but this is most likely to be a record review within health facilities, based on the project descriptions [21,22]. We did not identify any studies that reported the percentage of deaths to women of reproductive age that were due to maternal causes, or reported how the magnitude of association between key socio-demographic characteristics, facility type and maternal mortality had shifted with COVID-19. Moreover, none of the studies reported data to calculate the maternal mortality rate.

**Table 1.** Study description for each included study

Reference	Study setting	Study design	Study population	Study dates	Outcomes reported
**Lumbreras-Marquez *et al*. 2020 [**18**]**	Mexico; country-wide	Review of weekly epidemiologic reports from the Mexican Ministry of Health	All live births and maternal deaths reported to Mexican Ministry of Health	**Pre-COVID-19 era**: 2011–2019 data available; 2019 extracted for this review **COVID-19 era**: 1 January 2020–9 August 2020	Maternal mortality ratio (per live births only) Causes of maternal death
**Gianella *et al*. 2021 [**19**]**	Peru; country-wide	Review of data from the national death registry information system	All maternal deaths registered in the registry information system	**Pre-COVID-19 era**: 2019 **COVID-19 era**: 1 January 2020–28 November 2020	Maternal mortality ratio (per live births only) Causes of maternal death
**Bell *et al*. 2020 [**15**]**	Uganda; country-wide	Review of data extracted from Health Management Information System	Pregnant/postpartum women who had their data recorded in the Health Management Information System	**Pre-COVID-19 era**: 2019 **COVID-19 era**: March 2020	Maternal mortality ratio (per all deliveries) *[study authors provided denominator data for this review]*
**Pattinson *et al*. 2020 [**16,17**]**	South Africa; country-wide	Review of data extracted from the District Health Information System	All women who delivered in public hospitals	**Pre-COVID-19 era**: Jan 2019-March 2020 **COVID-19 era**: April-December 2020	Maternal mortality ratio (per live births only) Maternal mortality ratio (per all deliveries)
**Shikuku *et al*. Pre-publication [**20**]**	Kenya; country-wide	Review of data extracted from Kenya Health Information System	Pregnant/postpartum women who had their data recorded in the Kenya Health Information System	**Pre-COVID-19 era**: March to June 2019 **COVID-19 era**: March to June 2020	Maternal mortality ratio (per live births only) Maternal mortality ratio (per all deliveries)
**Kumari, Mehta and Choudhary, 2020 [**21**]**	Western India; four facilities in an integrated tertiary care medical college	*Unclear: probably facility record review*	Pregnant women attending any of the study facilities	**Pre-COVID-19 era**: 15 January 2020 to 24 March 2020 **COVID-19 era**: 25 March 2020 to 2 June 2020	Maternal mortality ratio (per all deliveries)
**Goyal *et al*. 2021 [**22**]**	Jodhpur, India; Department of obstetrics and Gynaecology, All India Institute of Medical Sciences	*Unclear: probably facility record review*	All pregnant women admitted to facility during study period	**Pre-COVID-19 era**: 1 October 2019 to 29 February 2020 **COVID-19 era**: 1 April 2020–31 August 2020	Maternal mortality ratio (per all deliveries)

### Risk of bias assessment

All of the studies were judged to be at high risk of bias for at least one of our assessment criteria, as shown in Table 2. The two studies from India were considered at high risk of bias with respect to their geographical coverage of the country [21,22], with all other studies judged to be at low risk of bias as they were national [15,17–20]. None of the studies were judged to be at low risk of bias in the extent to which the data sources captured both facility and home deliveries: five of the studies only captured institutional deliveries and therefore were considered high risk of bias for this criterion [15,17,20–22]. For the studies in Mexico and in Peru, it was unclear the extent to which they would capture births and maternal deaths that occurred at home [18,19]. The Peru study provided comparative estimates of maternal mortality from the Ministry of Health noting these to be higher than the national death registry information system which was utilised for the analysis, but found that the trends did not vary between these data sources [19].

**Table 2.** Risk of bias for each study quantifying the impact of COVID-19 on maternal mortality

Reference	Extent to which estimates represent the country geographically	Extent to which estimates represent facility & home deliveries/deaths	Definition of maternal death	Definition of denominator(s)	Classification of pre-COVID & COVID-19 time periods	Comparability of pre- and post-COVID-19 study populations
**Lumbreras-Marquez *et al*. 2020 [**18**], Mexico**	The source of data is clearly stated as Mexican Ministry of Health reports, so geographically comprehensive	In theory, these reports should capture deaths and births that occur in facilities and home; however, the authors provide no information on the extent to which this is the case	Authors note that they capture deaths from all causes – which suggests they are measuring pregnancy-related mortality rather than maternal mortality	Clearly defined as live births	The early part of 2020 is included in COVID-19 time period, despite the first confirmed cases of COVID-19 on the 28 February 2020 and entering strict lockdown on 30 March 2020 in Mexico	Average monthly number of lives births captured in reports was lower in 2020 (~176,213) to 2019 (~184,887) but this difference is <10%.
	**Low**	**Unclear**	**High**	**Low**	**High**	**Low**
**Gianella *et al*. 2021 [**19**], Peru**	Nationally representative – national death registry information system	In theory, this data source should capture deaths and births that occur in facilities and home; however no data in provided on completeness aside from noting that many deaths in the national death registry system are missing cause, and were therefore excluded, and much lower levels of maternal mortality when compared with data from the Ministry of Health	Clearly stated as any women between age 12 and 57 that had at least one cause of death labelled as “pregnancy, childbirth and postnatal” (ICD-10 codes O00-O99)	Clearly defined as live births	The early part of 2020 is included in COVID-19 time period, despite the first confirmed cases of COVID-19 on the 7 March 2020 and entering strict lockdown on 30 March 2020 in Peru	Average monthly number of live births captured in reports was similar in 2020 (~39,037) to 2019 (~40,686)
	**Low**	**Unclear**	**Low**	**Low**	**High**	**Low**
**Bell *et al*. 2020 [**15**]**, **Uganda**	Nationally representative – Health Management Information System data	The Health Management Information System data will only capture facility information; not clear whether the coverage of different facility types is comprehensive	No information on the definition of maternal death	Clearly defined as all deliveries (data made available from authors)	For the “COVID-19 period”, data are only available for March 2020 which is only likely to capture limited impacts of the COVID-19 pandemic given the first case was reported in Uganda on the 21^st^ March, with social-distancing policies from March 12^th^	Fall in institutional deliveries from an average of 99,484 per month pre-COVID-19 to 71,489 in March 2020; if women who attended the facility during COVID-19 are very different to those who did not attend then will lead to bias in maternal mortality ratio
	**Low**	**High**	**Unclear**	**Low**	**High**	**High**
**Pattinson *et al*. 2020 [**16,17**], South Africa**	South Africa has a routine data system (the District Health Information System) that collects data on the usage and outcome of the various services on offer in the public sector, and covers the whole country	Only covers institutional events within the public sector	No information on the definition of maternal death	Clearly defined as livebirths and all deliveries	COVID-19 time period starts from April 2020 which is likely to minimise misclassification given the first case was identified in South Africa on 5 March 2020 and the country entered strict lockdown on 19 March 2020	No evidence for a reduction in the average number of deliveries that occur in facilities pre-COVID-19 (average number of deliveries per quarter: 244,432) and since COVID-19 (average number of deliveries per quarter: 252,543)
	**Low**	**High**	**Unclear**	**Low**	**Low**	**Low**
**Shikuku *et al*. Pre-publication [**20**]**, **Kenya**	Health Management Information System data which covers the whole country	The Health Management Information System data will only capture facility information; not clear whether the coverage of different facility types is comprehensive	No information on the definition of maternal death	Clearly defined as either total deliveries or live births only	COVID-19 time period starts from March 2020 which is likely to minimise misclassification given the first case was identified in Kenya on 13 March 2020 and the country entered strict lockdown in April	Total number of deliveries recorded increased from 394,852 in pre-COVID-19 time period to 398,538; it is possible that different facilities reported to HMIS system between time periods
	**Low**	**High**	**Unclear**	**Low**	**Low**	**Low**
**Kumari, Mehta and Choudhary, 2020 [**21**]**, **India**	Four hospitals in an integrated tertiary care medical college in Western India	Only captures maternal mortality in tertiary level facilities	No information on the definition of maternal death	The authors describe the numbers of women admitted, which is assumed to be for labour management (which is also referred to in paper) but not completely clear.	Use date of lockdown to define pre-COVID-19 and COVID-19 time periods	Dramatic fall in number of pregnant women hospitalised [for labour management] at these four tertiary hospitals, from 6209 to 3527 for pre COVID-19 and COVID-19 periods, respectively; note that women who deliver in study facilities after lockdown were more likely to be literate and primigravidae compared to women delivering pre-COVID-19, and there were fewer obstetric emergencies
	**High**	**High**	**Unclear**	**Unclear**	**Low**	**High**
**Goyal *et al*. 2021 [**22**]**, **India**	A single centre in India	Only captures maternal mortality in a tertiary level facility	No information on the definition of maternal death	Pregnant women admitted to the Department of Obstetrics	COVID-19 time period starts from April 2020, which is the first full month following lockdown (implemented in mid-March) and therefore little risk of misclassification in pre-COVID-19 and COVID-19 time periods	Dramatic fall in institutional deliveries from 1062 to 633 for pre COVID-19 and COVID-19 periods, respectively; note that the percentage of high risk pregnancies increases from 45.2% pre-COVID-19 to 52.4% since COVID-19
	**High**	**High**	**Unclear**	**Low**	**Low**	**High**

The study from Peru provided a clear definition of a maternal death and was considered at low risk of bias [19]. Of the remaining six studies, one study suggested that deaths from any cause during the pregnancy and postpartum period were included [18] and five studies provided no detail of their definition of maternal death [15,17,20–22]. The denominator was clearly defined as all births, deliveries or live births in six of the studies [15,17–20,22]. The final study was considered at unclear risk of bias, as the authors describe the number of women admitted and separately imply this is specifically for labour management [21].

The definition of the pre-COVID-19 and COVID-19 exposure periods varied substantially across the different studies. In five of the studies, the COVID-19 period was defined from when there was likely to have been impact of COVID-19 (i.e. following the first confirmed cases or lockdown); four of these studies were considered at low risk of bias for this criteria [17,21,22], with the final study still classified as at high risk of bias as there was only a few weeks of data included in the COVID-19 period [15]. The remaining two studies were considered at high risk of bias as they included the start of 2020, before there were any confirmed cases or any mitigation measures in place, as part of the COVID-19 period [18,19].

There was no evidence for a decrease in the average number of deliveries or live births in four of the studies [17–20], and these studies were considered at low risk of bias for comparability of populations between the pre-COVID-19 and COVID-19 time periods. However, the other three studies were considered at high risk of bias as they documented substantial decreases in the number of deliveries captured after the onset of COVID-19 [15,21,22] so affecting comparisons in the maternal mortality ratio over time owing to changes in the denominator. Two of these studies provided further details, with one noting that the study population in the COVID-19 period were more likely to be literate and primigravidae, and there were fewer referred obstetric emergencies [21], and the other study documenting an increased percentage of high-risk pregnancies compared to the pre-COVID-19 period [22].

### Study results

**Figure 2.**
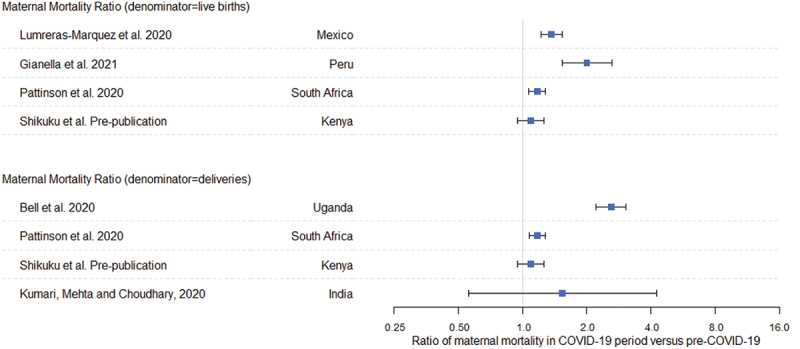
Ratio of maternal mortality ratio since COVID-19 to the maternal mortality ratio in the pre-COVID-19 time period; Goyal et al. study excluded as no maternal deaths in pre-COVID-19 period [22].

Figure 2 and Table 3 show the key results of the seven included studies. There were increased levels of maternal mortality documented in all studies relative to the pre-pandemic period; however, there was only statistical evidence for a difference in maternal mortality in the COVID-19 era for four of the studies [20–22] (Figure 2).

**Table 3.** Study results for each included study

Reference	Time period	Number of maternal deaths	Number of live births	Maternal mortality ratio (per 100,000 live births) [MMratio-LB]	Number of deliveries	Maternal mortality ratio (per 100,000 deliveries) [MMratio-D]	Change in maternal mortality ratio with COVID-19
**Lumbreras-Marquez *et al*. 2020 [**18**]**, **Mexico**	**Pre-COVID-19 era**: 2019	690	2,218,649*	31			26.2%
	**COVID-19 era**: 1 January 2020–9 August 2020	523	1,233,491*	42			
**Gianella *et al*. 2021 [**19**]**, **Peru**	**Pre-COVID-19 era**: 2019	83	488,235*	17			50.0%
	**COVID-19 era**: 1 January 2020–28 November 2020	146	429,412*	34			
**Bell *et al*. 2020 [**15**]**, **Uganda**	**Pre-COVID-19 era**: 2019	1074			1,193,805	90	61.5%
	**COVID-19 era**: March 2020	167			71,489	234	
**Pattinson *et al*. 2020 [**16,17**]**, **South Africa**	**Pre-COVID-19 era**: January 2019-March 2020	1190	1,197,247	99	1,222,158	97	15.4% (MMratio-LB) 14.9% (MMratio-D)
	**COVID-19 era**: April-December 2020	866	742,957	117	757,629	114	
**Shikuku *et al*. Pre-publication [**20**]**, **Kenya**	**Pre-COVID-19 era**: March to June 2019	373	385,996	97	394,852	94	8.5% (MMratio-LB) 8.7% (MMratio-D)
	**COVID-19 era**: March to June 2020	412	389,437	106	398,538	103	
**Kumari, Mehta and Choudhary, 2020 [**21**]**, **India**	**Pre-COVID-19 era**: 15 January 2020 to 24 March 2020	8*			6209	129	35.8%
	**COVID-19 era**: 25 March 2020 to 2 June 2020	7*			3527	198	
**Goyal *et al*. 2021 [**22**]**, **India**	**Pre-COVID-19 era**: 1 October 2019 to 29 February 2020	0			1062	0	-
	**COVID-19 era**: 1 April 2020–31 August 2020	2			583	343	

*Extrapolated from percentages/rates reported in paper; MM = Maternal Mortality; LB = Live births; D = deliveries

Two studies reported the maternal mortality ratio using live births as the denominator, showing increases in the maternal mortality ratio in the COVID-19 era compared to the pre-COVID-19 period of 50.0% in Peru [19] and 26.2% in Mexico [18] (Table 3). Three studies reported data to calculate the maternal mortality ratio using all deliveries as the denominator, although there were no deaths observed in the pre-COVID-19 era for one of the studies from India [22]. For the other two studies, the maternal mortality ratio increased by 61.5% in Uganda [15] and 35.4% in India in the COVID-19 era [21]. Two of these studies reported data such that the maternal mortality ratio could be calculated using either live births or all deliveries as the denominators [20]. In South Africa, the mortality ratio estimated using live births increased by 15.4% compared with 14.9% based on using all deliveries as the denominator [16], and in Kenya, the equivalent estimates of the increase in maternal mortality were 8.5% and 8.7%, respectively [20]. The study from South Africa also reported 30% excess maternal deaths when comparing exactly the same months (April to September) in the pre-COVID and during COVID periods [17].

Two studies reported information on cause of maternal death [18,19]. In Peru, it was noted that the percentage of maternal deaths which had pre-eclampsia or eclampsia listed as the principal cause of death increased in 2020 [19], and that 24% of maternal deaths in the pandemic period were categorised as COVID-19 cases. In the study from Mexico, the authors provided sufficient data to calculate the cause-specific maternal mortality ratios per live births [18]; there was a substantial increase in maternal deaths due to respiratory disease (from 1.7 per 100,000 live births in 2019 to 13.6 per 100,000 live births in 2020). The cause-specific mortality ratio per 100,000 live births increased slightly for hypertensive disorders of pregnancy (from 6.4 in 2019 to 6.7 in 2020) and for postpartum haemorrhage (from 6.4 in 2019 to 7.3 in 2020). Decreases were documented for venous thromboembolism (from 1.0 in 2019 to 0.3 in 2020) and for other causes (from 15.6 in 2019 to 14.4 in 2020).

## Discussion

Much has been written over the years about pregnancy and childbirth in the face of adversity – be this the Dutch famine of the 1940s, the ongoing humanitarian crisis in Yemen, or the Ebola epidemic in 2014–16 in West Africa [23–25]. The ability and desire to reproduce in such challenging circumstances has both physiological and socio-political significance. However, this drive may not be matched by the capacity of the health system to provide accessible, high-quality maternity care. Maternal deaths are a marker of this mismatch and rapidly reflect shocks to services. What does not respond so rapidly is the ability to capture these tragic events, and to differentiate between excess mortality due to physiological impacts of a health-related disaster, such as a pandemic or famine, versus those due to concurrent failure of the health system to respond.

One of the earliest papers exploring the impact of COVID-19 on maternal and child outcomes, developed three model scenarios with the least severe of these yielding an 8.3–38.6% increase in maternal deaths per month across the 118 countries [26]. Since then, there have been further headline projections of increases in maternal mortality [27,28], often linked to the alarming evidence accumulating on the collapse of maternity services and restrictions on movement, which have led to massive falls in uptake of care at the time of delivery [29]. Whilst logical to assume a consequent increase in maternal mortality, the level in a whole population as measured by the maternal mortality ratio reflects a balance of factors affecting the risk of death once pregnant, which varies between women due to many factors, such as health status, parity, age, socio-economic status and location. How a pandemic such as COVID-19 and the associated response measures impact differently on these risk differentials, and indeed on the level of fertility, is not only an important question for predicting trends but also crucial for identifying optimal mitigation strategies. While there is mounting evidence for falling fertility at a population level in many countries, during the COVID-19 pandemic [30], if and when this translates into an impact on pregnancy-related death – since, by definition, all such deaths are contingent on the occurrence of pregnancy in the first place, is uncertain. This means that research must consider the possibility that the level of maternal mortality may move in either direction – up or down. This was the starting point for our rapid systematic review.

The review was first conceived when the pandemic had been in place for over 6 months and when an exponential rise in the conducted studies and published articles was observed. A reasonable yield of papers on this important topic and insights on many of the influencing factors was therefore expected. However, only seven papers met the selection criteria and provided data on the main outcomes, out of a total of 3411 initial articles. This 0.2% yield is low by any standards, and becomes miniscule when compared with the magnitude of the overall publication database since COVID-19 emerged. Our review was not restricted geographically and the lack of papers on levels of maternal mortality is applicable globally, including to high-income settings where mortality surveillance is well established and additional reporting systems were also frequently setup. There are many reasons why these data may not have been identified in our search where they exist, most notably a prioritisation of national-level exploration of any available data to inform local responses without making it publicly available and the time-scales for peer-review and publication of articles. Interestingly, all included papers came from low- and middle-income countries (LMIC), where high-quality, comprehensive routine reporting systems are often lacking. One of the most frequent reasons for exclusion were papers which only discussed maternal mortality in COVID-19-related cases without any comparison group [31] or without a comparison with pre-COVID levels [32]. A rapid report from the UK, for example, identified 16 maternal deaths directly related to COVID-19 – either women with confirmed or suspected SARS-CoV-2 infection during or up to 1 year after pregnancy, or women who died from mental health-related causes or domestic violence – between March 2020 and May 2021, but did not report all maternal deaths for this period making it impossible to compare the pre- and post-pandemic levels of maternal mortality [33]. The focus on direct COVID-19-related maternal deaths in high-income settings could possibly imply a comparative neglect of the potential collateral mortality effects of the pandemic, both in data capture and in response strategies and mechanisms to protect maternity patients and services.

The seven studies all indicate an increase in maternal mortality compared to pre-COVID levels, although only four of these reached statistical significance and all have a high risk of bias in the data for at least one of our quality criteria. To our knowledge, this is the only systematic review which focuses specifically on levels of maternal mortality in pre-COVID and during COVID periods. An earlier living systematic review by Allotey and colleagues only included deaths in pregnant or recently pregnant women with suspected or confirmed COVID-19 [7], and the recent review by Chmielewska and colleagues included a wide range of outcomes [34], but only two studies contribute to their maternal mortality analysis. Both these reviews concluded that there has been an increase in maternal mortality but we would encourage greater nuancing to such conclusions [7,34]. Our own review has highlighted four important factors in the capture and reporting of maternal mortality, which limit drawing firm conclusions about trends, but do provide pointers to areas for improvement in future studies; we have integrated text panels below to provide further elaboration on these measurement issues, which also lie at the heart of Professor Peter Byass’s work.

Firstly, like any specific cause of death, the definition used is crucial to creating reliable estimates. Maternal death is precisely by WHO [14], and yet misclassification is common. Pregnancy-related death is a convenient complementary definition by encompassing all death to women during pregnancy or the postpartum period, which is especially relevant to settings where high-quality information on cause of death is rare. Five of the seven studies included in this review provided insufficient detail to establish the definition used and thus had to be categorised as ‘unclear’ in the quality assessment. It is crucial that future studies are explicit about the definitions adopted as well as the methods used to assign the cause, be this medical certification or verbal autopsy methods. The latter continues to play a crucial role in LMICs for many causes of death, and Panel 1 summarises the development of verbal autopsy for pregnancy-related mortality. With the recent emergence of SARS-CoV-2, WHO issued an emergency ICD code for use in cause of death reporting [35], and verbal autopsy methods have also been rapidly adapted to accommodate signs and symptoms linked to COVID-19. In line with the existing maternal classification, where a death was caused by SARS-CoV-2 and the disease was aggravated by the physiological effects of pregnancy, this is to be classified as a maternal death and specifically an indirect obstetric death. In terms of this review, notably only two studies gave information on cause-specific deaths, with a quarter of the deaths in Peru categorised as COVID-19 cases [19], and a stark increase in maternal deaths from respiratory disease in Mexico, which are likely to be COVID-19 related [18].

**Panel 1.** The development of verbal autopsy tools for pregnancy-related death

Measurement methods must be determined by local circumstances, pragmatism and intended use of the data – principles that are always important but perhaps even more critical during the COVID-19 pandemic. Notable work adopting this ethos was Professor Peter Byass’s adaptation of verbal autopsy (VA) methods to create a specialised Bayesian tool for interpretating VA data for deaths of women of reproductive age, known as InterVA-M [36]. The InterVA-M method applies Bayes’ theorem to calculate the likelihood of causes of death as well as the likelihood for each individual being pregnant at death, dying within six weeks of pregnancy ending, or not being recently pregnant, which is important in cases where pregnancy status at death may be ambiguous in the VA interview data. The approach recognises the messiness of VA procedures and uncertainty of symptom reporting by VA respondents and builds this into the probabilistic approach rather than letting it be a barrier to measurement. Preliminary development and testing of InterVA-M compared well to interpretation of VA data using physician review, but with the added advantages of speed and consistency [36]. This work also initiated new thinking on concepts of validity of cause of death in comparison to medical records or physician review of the same data, arguing for pragmatic comparisons in terms of comparability, reliability and adequacy of purpose. Subsequent application in a range of settings [37,38], including on handheld devices [39], and refinement and integration with the full InterVA method constructed to reflect the WHO VA instrument and compatible with International Classification of Diseases coding, resulted in new opportunities for wider implementation of routine cause-of-death registration, not only in research environments [40]. Further development of InterVA to include collection and processing of information on non-medical causes and circumstances surrounding death provides an opportunity to capture information on the dynamics of healthcare access, likely to be critical in understanding indirect effects of COVID-19 with potential to inform action-oriented strategies to address disadvantage and restricted access to life-saving care for mothers [41]. Whilst the world’s focus is currently on strategies to reduce the burden of COVID-19, such innovations in pragmatic, direct measurement are a step towards data processes that are embedded in and owned by local systems.

The second lesson from this rapid systematic review relates to the key question of population coverage and representativeness. An important distinction is between population-based – meaning all deaths regardless of location – and just those occurring in health-care facilities. With the impact of COVID-19 on disrupting service availability and travel to health care [42], comparison of trends pre- and during COVID-19 in the maternal mortality ratio is complicated by shifts in case-mix in the numerator (maternal deaths) and denominator (live births or deliveries). It is crucial for studies to be explicit about the population base of their estimates, and for reviews or syntheses not to pool population and facility-based estimates. Many LMICs and their Maternal Death Surveillance and Response systems have to rely on the latter as identifying and reporting of deaths that occur at home is a huge challenge. Five of the papers did not capture deaths outside of health-care facilities, including the one for South Africa and Panel 2 provides additional insights on the challenge of home-death reporting in this specific country. Thus, only two of the seven papers in this review reported population-based trends in levels of maternal mortality, although neither included information to gauge the coverage or representativeness of their national death registries. It is worth highlighting that Kumari *et al*. document a dramatic fall in the number of pregnant women hospitalised for labour management (from 6209 in the pre-COVID period to 3527 in the COVID period) [21]. Given the early stage of the pandemic, this reduction is likely to be largely due to women seeking care at different facility levels or staying at home for delivery. For those of us seeking to understanding the impact of COVID-19 on maternal mortality, this shift in service utilisation is potentially distorting the comparison of the maternal mortality ratio before and during COVID-19. It is possible, for example, that all low-risk women are going to lower-level facilities or remaining at home, in which case, the increase in the levels of hospital maternal mortality is related to the shifting case mix of women. Alternatively, the highest risk women may face more barriers to accessing care with COVID-19 (e.g. finding transport) and therefore, this might lead to an an underestimate of the maternal mortality ratio. We only have one part of the picture when looking at institutional data, which makes it very difficult to truly understand what impact COVID-19 is having on maternal mortality in the whole population.

**Panel 2.** Problems of measuring non-facility maternal deaths in South Africa

The National Committee for Confidential Enquiry into Maternal Deaths (NCCEMD) set up in 1998, is structured to report on facility deaths within the health system. This is reflected in its Saving Mothers reports which describe iMMR (institutional maternal mortality rate). Despite this, some non-facility deaths are reported to the NCCEMD. Families and indirect networks may report the death of a recently hospitalised or delivered woman to the health facility concerned which then notifies the death. Also, mortuaries in some provinces notify non-facility maternal deaths to the NCCEMD where evidence of current or recent pregnancy is found at autopsy. There is wide variation between provinces in the extent of autopsies performed due to shortages of forensic pathologists and not all are notified to the NCCEMD. The most recent triennial report described 3238 maternal deaths from 2017–2019, of which 101 (3.1%) occurred outside of a health facility [43]. Another mechanism for identifying non-facility maternal deaths is through vital registration (VR). The South Africa (SA) death certificate includes questions on location of death and current or recent pregnancy (within 6 weeks). Death certificates are completed by doctors, but when not available, traditional leaders may notify community deaths via an abbreviated death certificate. There is no systematic way of linking the NCCEMD and VR data by developing cross linkages; and there are problems with the quality of death certificate completion.
The VR data is processed by STATS SA (a governmental statistics unit). It is then further analysed by the Burden of Disease Unit at the Medical Research Council which groups deaths by ICD 10 code, which include maternal death codes. In 2016, a collaboration between this unit and the NCCEMD enabled the two systems to be correlated for data from 1999 to 2014 [44]. This identified a greater number of non-facility maternal deaths than the NCCEMD; 70% occurred in facilities, 18% outside facilities and for 12% location of death was not recorded. The pattern of cause was similar to that reported by the NCCEMD. Given that over 90% of births in SA are in facilities, it is likely that many of the postpartum home deaths occurred in women who had had a facility delivery. There are two Health and Demographic sites (HDSS) in SA in Agincourt and Hlabisa which perform community surveillance and employ verbal autopsy methods to identify non-facility deaths, including maternal, showing that up to 30% of the total were non-facility maternal deaths [45]. The integration of verbal autopsy into HDSS in SA owes much to Professor Peter Byass, who worked extensively with the Agincourt site for many years to ensure true population-based estimates of cause-specific mortality were available Given the problems in identifying non-facility deaths, it is not possible to investigate their trends during the COVID-19 pandemic, although a repeat of the collaboration conducted in 2016 would be of value. Facility births did not show significant change, suggesting that there was not a move to more home births. However, there were changes in provincial distribution of births with increases noted in several rural provinces. This suggests population movement during the restrictive lockdown in 2020 [17] with a consequent impact on denominators for iMMR

The third lesson relates to the importance of capturing key co-variates of maternal deaths. As noted earlier, this rapid systematic review sought to look at whether the association between socio-demographic characteristics and levels of maternal mortality had changed, but none of the seven studies reported such information. Where the burden of excess mortality falls in terms of sub-groups of women is thus unknown. Information on women’s characteristics are often limited in routine data systems (including both vital registration, and health and management information systems), but such details are crucial to explore differentials and to design equitable interventions. One important resource in some LMICs which has greatly increased the availability of detailed individual-level socio-demographic and health information is Health and Demographic Surveillance Systems (HDSS), as summarised in Panel 3 which highlights their potential for measuring the impact of COVID-19. For maternal mortality in particular, HDSS can show the proportion of facility- versus home-based maternal deaths, as noted earlier in Panel 2, and so reveal important shifts in place of death in the face of the pandemic. Such special systems of course require continuous investment to sustain their key features of total population coverage and longitudinal measurement, and many have to rely on research funding so do not represent a competitor but rather a complement to strengthening routine information systems.

**Panel 3.** Counting maternal deaths in the COVID-19 era: the importance of health and demographic surveillance sites

This paper has highlighted an important gap in the empirical data on maternal mortality in the COVID-19 era; a lack of population-based data capturing births and maternal deaths that occur outside of health facilities. Civil and vital registration systems (CVRS) are very useful for providing national-level information on all births and deaths within a country, but recent estimates suggest that only 73% of countries have a comprehensive system capturing at least 90% of births and 68% a system that captures at least 90% of deaths [46]. Unsurprisingly, it is largely countries with relatively low levels of maternal mortality that have data from CVRS available. In the absence of CVRS, many countries have instead relied on data from health and demographic surveillance sites (HDSS) which collect detailed longitudinal data on geographically defined populations, including on births, death and migrations in the study population. HDSS have been widely used to quantify levels and causes of maternal death [47–49], although not without challenges. The frequency of data collection and the reliance on information reported for all household members by a household head has been found to impact on the quality of data on pregnancies and their outcomes [50,51]. Moreover, many of the HDSS cover relatively small geographical areas, leaving such individual HDSS unable to document changes in rare outcomes – such as maternal deaths – rigorously. The establishment of networks of HDSS, of which Professor Peter Byass was key player, have facilitated both comparative and pooled analyses of mortality in pregnant and postpartum women across Asia and Africa, overcoming some of the challenges posed by the small sample sizes [52]. By drawing on data from regular population-based HIV surveys across six HDSS, we were able to document that women living with HIV were nearly eight times more likely to die during the pregnancy and postpartum period compared with their uninfected counterparts [53]. Given their geographical reach, population-based coverage and wealth of historical data on levels and causes of mortality as well as on births, HDSS are uniquely placed to help us to understand the impact of COVID-19 pandemic on levels and causes of maternal mortality in some of the highest burden countries. There are already examples of HDSS which have adapted their data collection procedures in response to COVID-19 by, for example, conducting data collection over the phone rather than through household visits, and embedding screening for COVID-19 in their surveys [54]. Professor Byass repeatedly highlighted the importance of empirical data from HDSS [55,56], and our paper is a timely reminder of the value of these data.

Finally, the conduct of this rapid systematic review has revealed the need for an explicit standard for reporting maternal mortality, both to ensure essential information is provided to assess quality and risk of bias, and to increase the opportunity for the empirical data to be usable for global and national estimation processes, such as that undertaken by the UN Maternal Mortality Estimation Interagency Group (MMEIG) as described in Panel 4. As can be seen, the MMEIG acts as the international body striving to improve the availability and quality of data on maternal mortality to enable comparisons between countries and regions and over time in terms of progress towards development targets, including the use of modelled estimates. Inconsistencies in reporting make such comparisons challenging, and affect interpretation of findings from systematic reviews as demonstrated by this paper and highlighted in Table 2. A standard reporting form to capture maternal mortality and birth outcomes, along the lines of STROBE [57], would not only help ensure key aspects of data capture are declared, but may also help improve the quality of studies.

**Panel 4.** The challenges of producing global estimates

The UN Maternal Mortality Estimation Interagency Group (MMEIG) estimates are produced to be comparable internationally for global health monitoring, such as tracking progress towards the Sustainable Development Goals (SDGs). From a methodological standpoint, there are two key challenges to achieving comparable estimates, both arising from the input data: incompleteness & misclassification. Incompleteness refers to the extent to which deaths are unregistered (or ‘missing’); whilst misclassification refers to the extent to which an incorrect cause of death is assigned. How the MMEIG currently accounts for these depends on the data source [58]. Where data originates from a specialised study triangulating different sources, such as a confidential enquiry or Reproductive Age Mortality Study (RAMOS), and thus empirical data describing sensitivity and specificity is available, this observation can be included directly into the MMEIG Bayesian model. Where data originates from civil registration and vital statistics (CRVS) a separate Bayesian model is used as a first step to estimate a country-year specific adjustment factor, making use of the sensitivity and specificity data reported from specialised studies [59]. Where the data originates from another type of source: first, the observed proportion of maternal deaths among deaths of all women of reproductive age is used in preference to the maternal mortality ratio (MMR), the former being less influenced by incomplete reporting than the latter assuming non-differential reporting by cause of death; second, a 10% upward adjustment factor is applied. A generic adjustment factor is not ideal, and the MMEIG encourages countries to conduct empirical studies to quantify the extent of incomplete and misclassified maternal deaths so that their MMR can be better informed by national data. The prioritization of countries’ own data was a frequent call of Professor Peter Byass, who was a member and chair of the Technical Advisory Group to the MMEIG for many years. It is not yet known how the COVID-19 pandemic will affect the completeness of maternal mortality data: challenges including restrictions on movement, closure and remote working procedures within CRVS offices, and the inability for maternal death review committees to meet in-person have put systems under pressure and caused delays. Although this pandemic may bring specific challenges which are yet to emerge, there is evidence from other situations where information systems have been put under stress to show lasting adverse effects [60]. Certainly during the COVID-19 pandemic, there are restrictions on household surveys and other survey efforts which limit data availability. Countries are currently examining their 2020 data to ensure that they report the most complete numbers possible. Co-ordination between the various UN agency groups producing the global estimates, which often use estimates and data that are co-dependent on each other is of utmost important. Frequent discussions are taking place to take methodological decisions based on emerging and best available evidence. It is essential that an accurate underlying cause of death is recorded which will allow the disaggregation of maternal mortality due to SARS-COV-2, and excess maternal mortality due to disruptions in access to and/or quality of care. If the death was caused by COVID-19, and the COVID-19 was aggravated by the physiologic effects of pregnancy, then the death is classified as an indirect obstetric (maternal) death [14,61]. Attention should be paid to avoid the term ‘indirect’ when referring to excess maternal mortality, as this has the potential to create confusion. The importance of continued capacity strengthening to ensure the rules and standards are applied consistently cannot be understated.

The limitations of this rapid systematic review include the restrictions of the time period for inclusion of studies. We covered studies published between 1 January 2020 and 1 March 2021 only, and in a limited number of databases. We included pre-peer review and grey literature, which will not have been through an academic peer review process; however, on balance we felt it was important to capture as much data as possible given concerns that relevant data for this review were likely to be presented outside of journal articles. Whilst we tried to capture as much data as possible, it is likely that we missed relevant reports and presentations particularly if they were not published in English, and certainly if they were not made publicly available online. For some studies, it would have been possible to allow for possible seasonal patterns in deliveries and mortality by restricting to the same period to one full calendar year earlier, but we decided the longest period possible was preferable. The review had no restrictions on language, but it is possible that studies in the non-English language may have been missed. Given the heterogeneity between the studies included, the review did not undertake meta-analysis. Our risk of bias criteria required some arbitrary cut-offs and judgements to classify papers as high or low risk of bias. For example, for the domain classifying studies by the time point which they considered the COVID-19 period, we classified any study that included time before the date of the first case of COVID-19 or lockdown in their COVID-19 period as at high risk of bias. We acknowledge that there will have been some impacts of COVID-19 on, for example, health-care utilisation leading up to the first case of COVID-19 and lockdown in some settings, but all studies classified as at high risk of bias for this reason included data from the beginning of 2020 and so are almost certainly underestimating the impact in the early period. Additionally, when assessing the extent to which studies were geographically representative of the country, we made the judgment based solely on whether the data collection system was national, regional or facility based without understanding the completeness of these systems (which was not reported in any of the studies). It is certainly plausible that the national data systems may omit data from certain regions, and should have been classified as at high risk of bias. Despite these limitations, all seven studies indicated an increase in maternal mortality levels between the pre-COVID and COVID period, although our interpretation is cautious and supports the need for further studies and the use of standardised reporting. There is also a case for replicating this review as the pandemic continues to develop and as more data become available on maternal deaths and also on levels of fertility which ultimately drive levels of obstetric risk.

## Conclusion

Surveillance of mortality is a core component of outbreak response, and the COVID-19 pandemic has been unprecedented in prompting the set-up of new, or enhancement of existing, information systems in high, middle and low-income settings [62]. The mortality outcomes of concern are both those due directly to SARS-CoV-2 as well as deaths owing to the collateral or side effects of the pandemic related to pressures on health services as well as broader shocks, such as national lockdowns and economic hardships. Maternal mortality is an exemplar of both these effects, and tracking changes in levels from before and during the period of COVID-19 can help inform mitigation and recovery strategies for maternal and newborn health as well as other health outcomes.

This systematic review has flagged a dearth of published studies reporting on the impact of COVID-19 on levels of maternal mortality, and has highlighted a need to improve standards of reporting. We identified just seven relevant studies published since the onset of the pandemic. All of these suggest an upward trend in the maternal mortality ratio, supporting the hypothesis from Goodburn and Campbell that this outcome is a sensitive marker of health system functioning [63], and highlighting the challenges that – often already overstretched – health-care services have faced in providing essential care whilst dealing with the impact of COVID-19. It is, however, also important to note that only two of the studies are reported as covering all deaths in the population rather than just those occurring in health-care facilities. Interpreting changes in institutional maternal mortality ratios is challenging when data are only included from a subset of health-care facilities and given the dramatic change in maternity services that has occurred with COVID-19 across many settings [29]. We have outlined a number of recommendations on future reporting, and want to reiterate here the importance of studies reporting on the representativeness of their data and, where possible, the differential impact by key sociodemographic characteristics of women. The impact of COVID-19 is not borne equally within populations, and we must seek to understand how this influences maternal mortality. The importance of tracking the survival and well-being of all women and their newborns in the face of this pandemic cannot be overstated. Improved and increased data on maternal mortality can guide current responses as well as future rebuilding of equitable maternity care [34].

## Supplementary Material

Supplemental Material

## Paper context

The impact of COVID-19 on maternal mortality remains uncertain. In this review, we identify seven studies measuring maternal mortality during the COVID-19 period compared to pre-COVID-19, all of which suggest an upward trend. However, drawing firm conclusions was challenging due to methodological differences and weaknesses in the included studies. High-quality data on the impact of COVID-19 on maternal mortality are urgently needed to inform health systems response to the ongoing COVID-19 and to future pandemics.
