# Supplementary material for: Maternal mortality in the covid-19 pandemic: findings from a rapid systematic review
Source: Glob Health Action. 2022 Apr 4;14(Suppl):1974677. doi: 10.1080/16549716.2021.1974677 (PMC8986253; doi:10.1080/16549716.2021.1974677)
Supplement: Supplemental Material [file ZGHA_A_1974677_SM6870.docx]

Supplementary Table 1: Risk of bias assessment criteria

| **Domain** | **Risk of bias** |
| --- | --- |
| **Extent to which estimates represent the country geographically** | **Low risk of bias** if study should capture deaths across the whole country (Note that data completeness not considered but reported separately where available)  **High risk of bias** if study only covers small geographical facility (e.g. one or several health facilities)  **Unclear risk of bias** if geographical spread of study not provided |
| **Extent to which estimates represent facility & home deliveries/deaths** | **Low risk of bias** if study includes both home and facility based deliveries/deaths  **High risk of bias** if study only includes facility based OR home based deliveries/deaths  **Unclear risk of bias** if it is not clear whether both facility and home deliveries/births would be included |
| **Definition of maternal death** | **Low risk of bias** if study includes maternal deaths (so direct obstetric and indirect deaths)  **High risk of bias** if study includes all pregnancy-related deaths, regardless of cause of death  **Unclear risk of bias** if no definition is provided for maternal death |
| **Definition of denominator(s)** | **Low risk of bias** if study includes clear definition of denominator for the maternal mortality ratio as live birth or pregnancy or delivery  **High risk of bias** if study includes an alternative denominator such as number of pregnant women admitted to facility  **Unclear risk of bias** if no definition is provided for the denominator |
| **Classification of pre-COVID & COVID-19 time periods** | **Low risk of bias** if the COVID-19 period only included data after the first case of COVID-19 or lockdown and this period extended at least two months  **High risk of bias** if time before the date of the first case of COVID-19 or lockdown was included in the COVID-19 period or if follow-up in the COVID-19 period was particularly short (<2 months)  **Unclear risk of bias** if study dates covered by pre-COVID-19 and COVID-19 periods are not provided |
| **Comparability of pre- and post-COVID-19 study populations** | **Low risk of bias** if no evidence of change or change less than 10% in the average number of live births/deliveries between the pre-COVID-19 period and the COVID-19 period  **High risk of bias** if evidence of change of >10% in the average number of live births/deliveries between the pre-COVID-19 period and the COVID-19 period  **Unclear risk of bias** if data not provided to calculate the average number of deliveries per month or per quarter in the pre-COVID-19 and COVID-19 period |
